# The Monument to Adam

**Published:** 1880-04

**Authors:** J. B. Chandler


### THE MONUMENT TO ADAM.

J. B. CHANDLER.

At this time when the public mind has been awakened, especially in Elmira, to the proper appreciation of our great common ancestor, it would seem not inappropriate to give words to a few thoughts on the subject.

I do not propose to enter into any discussion as to the literal correctness of the generally accepted account of Adam, his creation, fall, &c; nor to touch upon the scientific theories as to man's origin. I will merely state in the last connection, that being a man, and sharing as I do with my fellow mortals the pride of being a "lord of creation," I was much gratified a few days since in reading that some distinguished scientist in noting certain common attributes in the brains of men and monkeys, had promulgated a counter theory to Darwin's, viz:—that from man came the ape and not man from the monkey.

Now whilst my pleasure at this discovery is slightly tempered with mortification at the degeneracy of man in those days, nevertheless, it is certainly gratifying to think that in going back on ones genealogical tree a chimpanzee is not found at its root.

In my intercourse with artists and sculptors, whilst endeavoring to aid in the embodiment of an idea for the proposed monument to Adam, I have through personal intercourse, correspondence and some little research, unearthed many interesting facts on the subject.

I find that in addition to the Jewish version of the creation as related in Genesis, all nations have some tradition as to the first man. The Japanese account is very graphic and curious and smatters greatly of Greek and Roman mythology. They have also fairy tales in which are portrayed the gradual development of the finer senses of man, until he realizes through those senses the fact of his actual existence.

Singular as it may seem, two artists, one an acknowledged sculptor rating among the highest in his profession, the other a young man of great native talent yet making his first steps in art, have made sketches in clay for the proposed monument, neither aware of the other's work and neither, I think, ever having heard of the Japanese fairy tale, yet both have seized upon this same idea though embodying it in different forms—the Japanese thought "the exact moment when Adam realizes that he exists."

Perhaps some of your readers may have shared the opinion frequently expressed as to the erection of a monument in Elmira to Adam: Why to Adam, can not they find somebody more deserving ?

If our public parks are to be decorated by statues to the gods and goddesses of mythology, why should not we call in to existence a "thing of beauty" which whilst "being a joy forever" will speak to us of reality and not of myths ?

Adam, made of clay, (as his name in Hebrew implies,) seems particularly a fit subject for monumental perpetuation. To us he marks the first mortal created, a perfect man. The Source from which he emanated creates perfection at first, the seed may and does degenerate, but the origin is perfection.

Therefore we may believe that our model possesses all the attributes of symmetry, beauty, stature and strength. Let the sculptor collect all that ancient and modern Art have done in modeling the human form and then he must strive for something beyond them in every point.

Then the peoples of the world may come to Elmira, believing as they choose as to the divine or mythic origin of the first man—Christians and Jews seeking the first mortal who sprang into existence at the word of the Great Creator; Japanese in search of the offspring of Izanaglie and Izanamie; or the Darwinian scientist looking for the "counterfeit presentment" of the first result of monkey transition into perfect man; all may stand on common ground in gazing at the statue of the "first man."

Let the good work then go on and see that in the selection of sculptor and design such judgment be used that in future the "Adam of Elmira" will have its acknowledged place in Art as unmistakable as the Apollo Belvedere or the Venus de Medici.

☞ Send in your subscriptions at once.

